# Transcatheter Aortic Valve Replacement in Discordant Aortic Stenosis: The Time Is Now

**DOI:** 10.1016/j.jscai.2024.101297

**Published:** 2024-02-05

**Authors:** Vikrant Jagadeesan, Carlos Sanchez, Steven Yakubov

**Affiliations:** a West Virginia Heart and Vascular Institute, West Virginia University School of Medicine, Morgantown, West Virginia; b Structural Heart Division, Riverside Methodist Hospital, OhioHealth, Columbus, Ohio; c OhioHealth Research Institute, Columbus, Ohio

**Keywords:** aortic stenosis, transcatheter aortic valve replacement

The 2020 American College of Cardiology/American Heart Association guidelines endorse a class I recommendation for symptomatic severe aortic valve stenosis (AS) regardless of the subtype that spans classic high-flow high-gradient AS (stage D1), classic low-flow low-gradient (cLFLG) with reduced ejection fraction (EF, stage D2), and paradoxical low-flow low-gradient (pLFLG) characterized by preserved EF >50% (stage D3).^1^ The natural history of the D2 subtype has a poorer overall prognosis compared with D1 or D3, as has been corroborated by multiple studies. Prognostic studies in D3 have demonstrated less consistent findings.

Hachicha et al^2^ first described the pLFLG AS (D3) subtype in 2007, highlighting that the population is typically female, older, and more likely to have hypertension, diabetes, coronary artery disease, and atrial fibrillation. D3 is characterized by smaller left ventricular size, concentric remodeling, impaired diastolic filling, high valvulo-arterial impedance, reduced long-axis LV function, increased interstitial fibrosis, and low global longitudinal strain (GLS).^3^^,^^4^ Natural history data in D3 are mixed, mainly restricted to small prospective cohort studies. Clavel et al^5^ report a 1.7-fold increase in all-cause mortality and 2.1-fold increase in cardiovascular mortality in D3 compared with D1. Lancelotti et al^6^ report even worse outcomes in D3: a 4.3-fold higher risk of cardiac events and a 5-fold lower risk of overall 3-year survival. In contrast, Maes et al^7^ demonstrate better survival over a median follow-up of 28 months in D3 compared with D1 (48% vs 31%; *P* < .01). The D3 subtype is also less likely to be referred for aortic valve replacement (AVR) due to potential misclassification of AS severity that may be due to variations in echocardiographic measurements and/or unfamiliarity with pLFLG AS diagnostic criteria.

In this issue of *JSCAI*, Benck et al^8^ and Ullah et al^9^ tackle a relative evidentiary gap in transcatheter aortic valve replacement (TAVR) for LFLG AS. With respect to D2 or cLFLG AS pathology, Ullah et al report a greater incidence of major adverse cardiovascular events and higher mortality compared with D1 patients in a meta-analysis of 21 observational studies including over 17,000 patients. This is somewhat expected, paralleling the trends of natural history studies. Benck et al^8^, in a retrospective single-center study, report improved quality of life (QOL) metrics and EF at 1 year. Out of 634 patients analyzed, only 76 patients met D2 criteria. The need for new pacemaker implantation was higher, while postprocedural stroke, dialysis, bleeding, and mortality were less than expected compared with other LFLG analyses. While insightful, the small sample size and inconsistencies in other findings of the study propose more conjecture rather than dicta in D2 prognostic considerations undergoing TAVR. Kansas City Cardiomyopathy Questionnaire (KCCQ) scores were lowest in the D2 subtype, and all improved significantly across all subtypes, with postoperative KCCQ not statistically significant between subtypes. While the greatest delta KCCQ was in D2, this is more indicative of the “regression to the mean” phenomenon, where a lower baseline will likely show a larger absolute value change after an intervention. PCI was protective against 1-year mortality in this study, which is in stark contrast to contemporary larger studies that have demonstrated worse outcomes with percutaneous coronary intervention prior to TAVR. In addition, the highest 30-day mortality was in the D1 subtype despite higher baseline Society of Thoracic Surgeons risk scores in the D2 subtype. At 1 year, D2 had the highest mortality. These findings are likely the consequence of an underpowered analysis. Importantly, this begs the question of how to balance an increased mortality signal with an increased QOL signal at 1 year. In a discussion with an individual D2 patient centered around treatment options, how does one reconcile an improved 1-year QOL benefit that is tempered by a higher mortality risk? Nonetheless, for the purposes of an initial study demonstrating a QOL benefit beyond 30 days in D2, the authors begin a fruitful discussion in dire need of further study.

The issue of TAVR in pLFLG AS, or the D3 subtype, is more complex. Ullah and colleagues report in their meta-analysis that major adverse cardiovascular events outcomes were similar in D3 compared with D1. While the sample size is more robust, follow-up is limited to a median of 1 year. Existing data from the PARTNER 2 SAPIEN 3 registry report that increasing baseline grades of diastolic dysfunction before TAVR were associated with increased cardiovascular death and rehospitalization over 2 years of follow-up^10^; however, these patients were predominantly D1 patients who happened to have concomitant baseline diastolic dysfunction. If the heart failure with preserved EF (HFpEF) phenotype parallels that of D3, then HFpEF parameters must be evaluated in D3 patients undergoing TAVR in a dedicated fashion. For example, the presence of atrial fibrillation contributes 3 points to the H_2_FPEF score, the most of any other parameter. How do concomitant baseline markers of left atrial (LA) myopathy, such as LA diameter and/or LA volumetric indices impact TAVR outcomes? A small retrospective study demonstrated improved LA reservoir and conduit but not booster function after TAVR; the study was purely descriptive, not in a dedicated D3 group, and not tracked with outcomes.^11^ Finally, are we measuring the correct outcomes in the D3 population? HFpEF patients are known to value improvement in functional health status over longevity, a concept called time trade-off. No measures of QOL or functional status, which typically are the primary endpoints in HFpEF trials, are reported in this meta-analysis. This reflects the exact vernacular used in the D3 ACC/AHA guideline: AVR is recommended if AS is the most likely cause of symptoms.

The concept of cardiac damage has come into focus particularly in LFLG AS. Généreux et al^12^ classified cardiac damage into 5 stages. The presence of myocardial fibrosis is associated with adverse long-term outcomes and less improvement in left ventricular ejection fraction and KCCQ scores in patients undergoing AVR. Mortality increases by an approximate 45% relative increment with each escalating stage of cardiac damage. These stages are independently associated with cardiovascular death and rehospitalization within 1 year after valve replacement and can be the strongest indicators of prognosis independent of the Society of Thoracic Surgeons risk score. Although GLS can aid in the sensitivity of early myocardial dysfunction and is well validated, it is far from standard across different practice contexts. Cardiac magnetic resonance imaging is more sensitive for myocardial fibrosis but not as practical for widespread implementation in TAVR workup. Nevertheless, nuanced baseline assessment of cardiac damage has gained contemporary interest, utilizing advancements in multimodality imaging in TAVR prognosis and outcomes. Early assessment of the degree of myocardial damage may alter the timing of valve intervention, potentially encouraging earlier valve intervention. This has gained momentum recently with early TAVR trials underway investigating TAVR in moderate AS with concomitant evidence of myocardial damage/dysfunction.

Benck et al^8^ and Ullah et al^9^ continue to help inspire the right questions to ask in TAVR prognosis across all AS subtypes. The time is now for a multicenter, registry-based, prospective study dedicated to LFLG AS further stratified by D2 and D3 subtypes that is ultimately propensity-matched to the D1 subtype over long-term follow-up. Covariates to consider are LA mechanics, tissue Doppler relaxation parameters, GLS, right ventricular/tricuspid valvular data, and other potential novel indicators of myocardial dysfunction. These predictors must then be coupled with appropriately selected outcomes that encompass QOL and functional health status to more accurately risk stratify severe AS across all subtypes that undergo TAVR.
